# PTEN interacts with RNA polymerase II to dephosphorylate polymerase II C-terminal domain

**DOI:** 10.18632/oncotarget.27128

**Published:** 2019-08-13

**Authors:** Ata Abbas, Todd Romigh, Charis Eng

**Affiliations:** ^1^ Genomic Medicine Institute, Lerner Research Institute, Cleveland Clinic, Cleveland, 44195 OH, USA; ^2^ Taussig Cancer Institute, Cleveland Clinic, Cleveland, 44195 OH, USA; ^3^ Department of Genetics and Genome Sciences, Case Western Reserve University School of Medicine, Cleveland, 44116 OH, USA; ^4^ Germline High Risk Focus Group, Case Comprehensive Cancer Center, Case Western Reserve University School of Medicine, Cleveland, 44116 OH, USA; ^5^ Present address: Division of Hematology Oncology, Department of Medicine, Case Western Reserve University, Cleveland, 44106 OH, USA

**Keywords:** phosphatase and tensin homolog (PTEN), cowden syndrome, PTEN hamartoma tumor syndrome, RNA polymerase II, Pol II CTD dephosphorylation

## Abstract

Gene transcription is a highly complex and strictly regulated process. RNA polymerase II (Pol II) C-terminal domain (CTD) undergoes massive cycles of phosphorylation and dephosphorylation during the process of gene transcription. These post-translational modifications of CTD provide an interactive platform for various factors required for transcription initiation, elongation, termination, and co-transcriptional RNA processing. Pol II CTD kinases and phosphatases are key regulators and any deviation may cause genome-wide transcriptional dysregulation leading to various pathological conditions including cancer. PTEN, a well known tumor suppressor, is one of the most commonly somatically altered in diverse malignancies. When mutated in the germline, *PTEN* causes cancer predisposition. Numerous studies have demonstrated that PTEN regulates the expression of hundreds of genes, however, no mechanism is known so far. PTEN is a dual specificity phosphatase, using both lipid and protein as substrates. In the present study, we demonstrate that PTEN interacts with the RNA Pol II and that PTEN expression is inversely correlated with global phosphorylation of Pol II CTD. Furthermore, PTEN dephosphorylates Pol II CTD *in vitro* with a significant specificity for Ser5p. Interestingly, ChIP-seq data analysis revealed that PTEN globally binds to promoter proximal regions, and PTEN loss increases genome-wide Pol II Ser5p occupancy, suggest that PTEN is a Pol II CTD phosphatase. Our observations demonstrate an unexplored function of PTEN with the potential of global transcriptional regulation, adding a new dimension to somatic carcinogenesis and germline cancer predisposition.

## INTRODUCTION

Somatic *PTEN* (phosphatase and tensin homolog) alterations are one of the most commonly observed in diverse malignancies. Germline *PTEN* mutations cause Cowden and related syndromes, conferring a high risk of breast, thyroid and other cancers. PTEN, a well-studied tumor suppressor, regulates cell proliferation, growth and survival by antagonizing the PI3K-AKT-mTOR pathway [1, 2]. PTEN has dual specificity phosphatase activity, dephosphorylating both lipids and proteins [3]. PTEN shuttles in and out of the cytoplasm and nucleus, and exhibits tumor suppressor activities, possibly through both its phosphatase and non-phosphatase functions [4, 5]. PTEN interacts with a wide variety of nuclear factors [6–8] and is involved in DNA repair, recombination, genomic integrity and chromatin condensation [9–13]. Numerous studies strongly suggest that PTEN is also involved in transcriptional regulation, whether by direct or indirect regulation, a process whose precise molecular mechanisms are largely unknown [14–16].

Gene transcription and its regulation are one of the most intricate biological process involving a wide variety of factors interacting with RNA Polymerase II (Pol II) complex. Pol II is comprised of 12 highly conserved subunits. RPB1, the largest subunit of RNA Pol II complex, contains a C-terminal domain (CTD) comprising 52 tandemly repeated YSPTSPS heptad sequences. The CTD undergoes substantial phosphorylation and dephosphorylation cycles during gene transcription. The dynamic phosphorylation of serine residues at positions 2, 5, and 7 in CTD of RPB1 provide an interactive platform for various factors that regulate transcription initiation, elongation, termination as well as RNA processing [17]. A series of Pol II CTD kinases and phosphatases have been discovered [18] and the list is expanding. However, the knowledge of alterations in these factors in various physio-pathological conditions impacting on Pol II CTD modifications and genome-wide transcriptional regulation is limited.

Very recently, we reported that PTEN helps establish genome-wide Pol II pausing and re-distributes Pol II occupancy across the genome. Furthermore, it may impact Pol II pause duration, release, and elongation rate to enable precise gene regulation [19]. Another very recent observation by Steinbach et al revealed that PTEN binds to promoters and putative enhancer regions and alters the expression of hundreds of genes [20]. These two recent studies, for the first time, shed light on an under-acknowledged role of nuclear PTEN in global transcriptional regulation. However, there are still missing parts in the puzzle to fully understand a more direct and precise role of PTEN in regulating global transcription. Here, we sought to understand how PTEN may play a role in regulating transcription.

## RESULTS

### PTEN expression correlates with Pol II CTD phosphorylation level in cells

**Figure 1:**
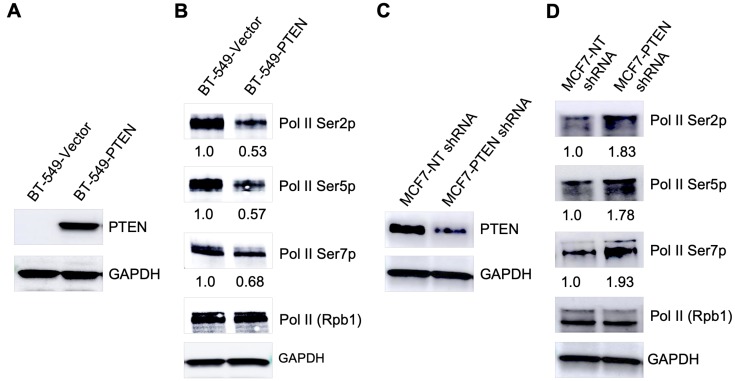
PTEN modulates global Pol II CTD phosphorylation. (**A**) Overexpression of PTEN in BT-549 (PTEN null) cell line. (**B**) Decrease in RNA Pol II CTD phosphorylation after overexpression of PTEN in BT-549 cells. (**C**) PTEN knock-down in MCF7 (PTEN-wildtype) cells. (**D**) Increase in RNA Pol II CTD phosphorylation after knock-down of PTEN in MCF7 cells.

Considering the dual phosphatase activity of PTEN, we wanted to know if PTEN dephosphorylates Pol II CTD to modulate genome-wide transcription. To test this hypothesis, we generated a stable PTEN-wildtype expressing line using BT-549 (PTEN null) breast cancer cells (Figure 1A). We found that overall phosphorylation of RNA Pol II CTD at the Ser2, Ser5, and Ser7 positions was decreased by 0.53, 0.57, and 0.68 folds respectively after exogenous expression of PTEN in BT-549 cells (Figure 1B). To confirm this observation in another cell line, we knocked-down of *PTEN* gene in MCF7 (PTEN-wildtype) breast cancer cells using shRNA (Figure 1C). Interestingly, a decrease in PTEN levels, after knock-down of *PTEN* in MCF7 cells, was associated with 1.83, 1.78, and 1.93 folds increase in CTD phosphorylation at Ser2, Ser5, and Ser7 positions respectively (Figure 1D). These observations using re-expression and knock-down models of PTEN in two different cell lines provide correlative evidence that Pol II CTD phosphorylation coincides with PTEN levels in breast cancer cells.

### PTEN interacts with RNA Pol II transcription machinery

**Figure 2:**
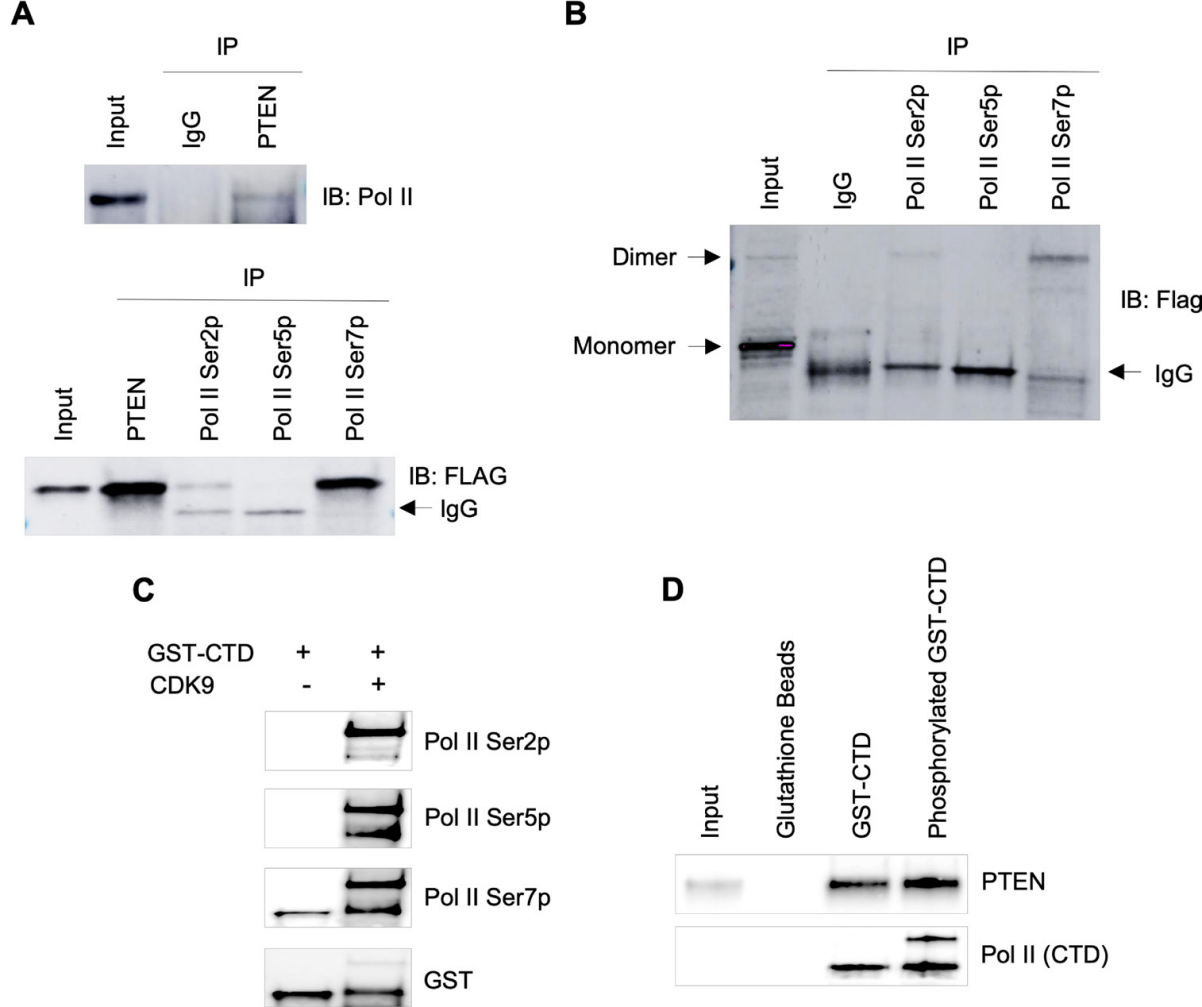
Association of PTEN with RNA Pol II transcription machinery. (**A**) Co-IPs from total lysate of BT-549 cells stably expressing PTEN 3x Flag. Top panel, pull-down with IgG and PTEN followed by Western blot with RNA pol II antibody (Rpb1). Bottom panel, IPs with PTEN, RNA pol II Ser2p, Ser5p and Ser7p antibodies. Western blot was performed using anti-Flag antibody. (**B**) Co-IPs from total lysate of BT-549-PTEN cells followed by Western blot under non-denaturing conditions, reveal dimers of PTEN. (**C**) CTD of RNA Pol II was phosphorylated by using CDK9+CyclinK for 2 hours at 30°C in a kinase buffer. (**D**) Pull down assay using phosphorylated or unphosphorylated GST-CTD tagged beads showing interaction of PTEN with Pol II CTD.

Next, we were interested to know if PTEN physically interacts with RNA Pol II. We performed co-immunoprecipitation using specific antibodies followed by immunoblotting. We detected PTEN binding to RNA Pol II, and this interaction may be affected by the phosphorylation status of specific Serine residues of Pol II CTD (Figure 2A). Interestingly, a small fraction of PTEN dimers was observed in the input lane, and that fraction was pulled down in co-immunoprecipitation (Figure 2B), suggesting PTEN interaction with Pol II might be in the form of a dimer. Furthermore, we used *in vitro* pull-down assay to re-confirm our findings. GST-tagged Pol II CTD peptide was phosphorylated using CDK9/CyclinT1 in a kinase reaction that resulted in Ser2, Ser5, and Ser7 phosphorylation of GST-CTD (Figure 2C). Using phosphorylated and unphosphorylated GST-CTD peptides, we performed PTEN pull-down assay and confirmed the interaction of PTEN with the CTD of RNA Pol II (Figure 2D). Considering control (unphosphorylated) GST-CTD peptides are slightly phosphorylated at Ser7 position (Figure 2C, lane 1) that would have contributed in PTEN pull-down, it became apparent that phosphorylated GST-CTD peptides have higher affinity to PTEN in comparison to unphosphorylated GST-CTD (Figure 2D).

### PTEN dephosphorylates Pol II CTD *in vitro*

**Figure 3:**
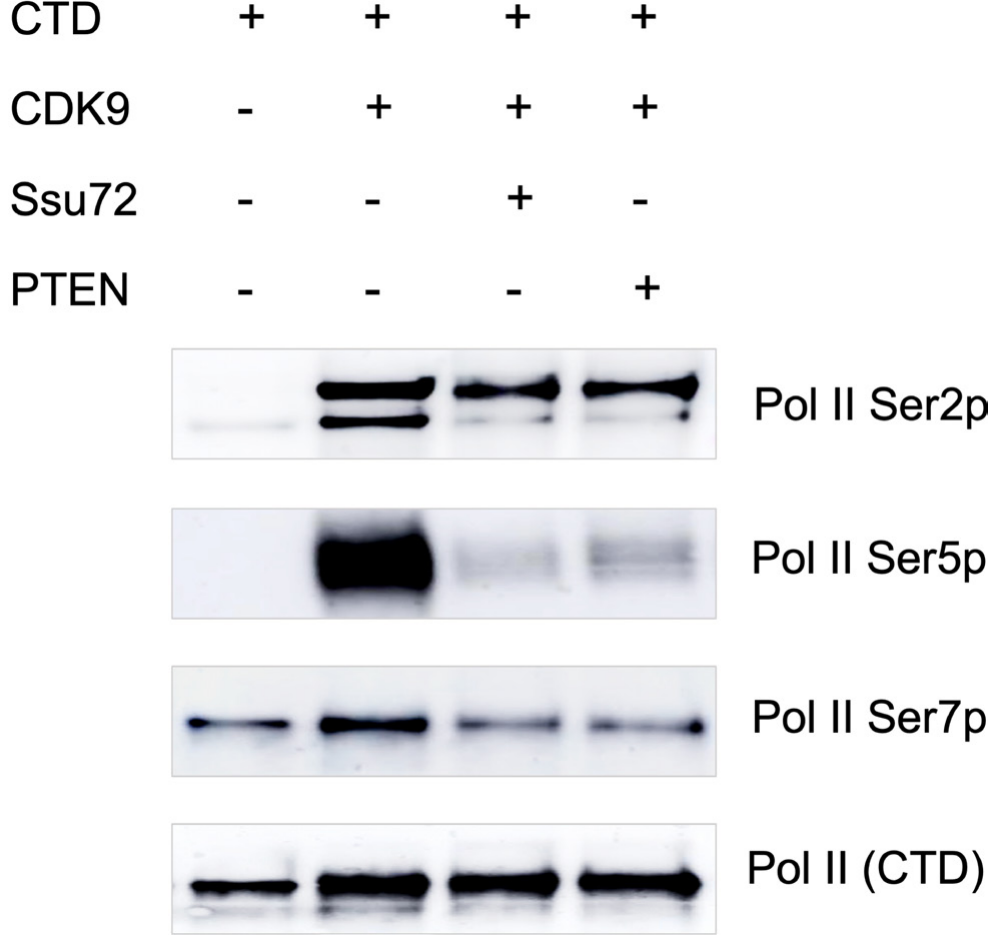
PTEN dephosphorylates Pol II CTD *in vitro*. 100 ng of CTD of RNA Pol II was phosphorylated by using CDK9+CyclinK for 2 hours at 30° C in a kinase buffer. Phosphorylated CTD was then treated with Ssu72, a known CTD phosphatase and PTEN followed by Western blotting with Pol II Ser2p, Pol II Ser5p, Pol II Ser7p, and Pol II CTD specific antibodies.

With the observation that PTEN-Pol II interaction and an association of global Pol II CTD phosphorylation with PTEN expression in cells, we asked the obvious question whether PTEN dephosphorylates serine residues of Pol II CTD. We performed an *in vitro* study using GST-tagged CTD peptide to understand if phosphorylated Pol II CTD is a substrate for PTEN phosphatase activity. First, the CTD peptide was phosphorylated using CDK9/CyclinT1 in a kinase reaction. Phosphorylated CTD was then used to test the phosphatase activity of purified PTEN. Ssu72, a known Pol II CTD phosphatase was also included as a positive control. Surprisingly, we observed a marked decrease in phosphorylation of Ser5 and Ser7, and a moderate reduction in Ser2 phosphorylation in Pol II CTD (Figure 3). We noted that *in vitro* results showing almost a complete dephosphorylation of Ser5p-CTD by PTEN. The rapid dephosphorylation of CTD at Ser5 position suggests that PTEN is a Pol II Ser5 phosphatase. This possibly explains the observation in Figure 2A showing the least pulldown of PTEN by Ser5p antibody in the co-IP experiments; however, further studies are needed to better understand the differential dephosphorylation kinetics as well as specificity of PTEN for various serine residues of Pol II CTD.

### PTEN loss increases Pol II Ser5 phosphorylation at genome-wide scale

**Figure 4:**
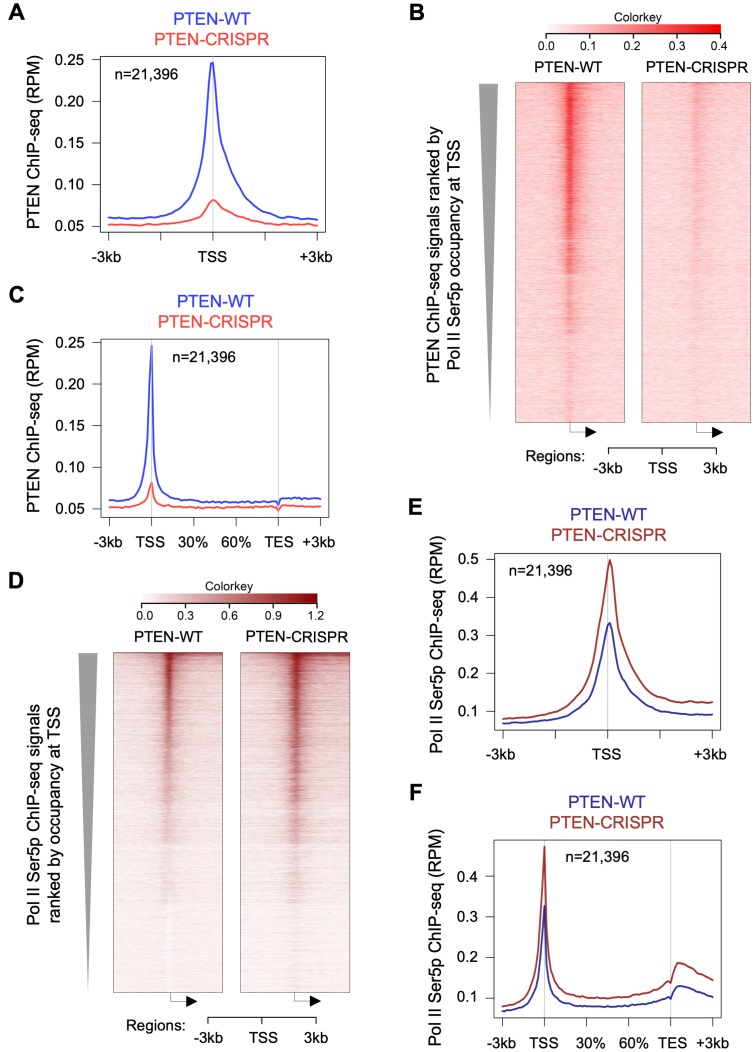
PTEN loss increases global Pol II Ser5 phosphorylation. ChIP-seq data were downloaded from GEO accession number GSE120439, and re-analyzed as described in Materials and Methods. (**A**) Metagene plot and heatmap (**B**) showing the occupancy of PTEN near TSS of 21,396 genes in HeLa cells (PTEN ChIP-seq datasets SRR7902981, SRR7902982, SRR7902987). (**C**) Metagene plot showing PTEN signals at gene bodies of 21,396 genes. (**D**) Heatmaps and (**E**) metagene plot showing Pol II Ser5p occupancy at TSS of 21,396 genes in HeLa cells (Pol II Ser5p ChIP-seq datasets SRR7902985, SRR7902990). (**F**) Metagene plot showing Pol II Ser5 occupancy at gene bodies of 21,396 genes. Gene list, containing 21,396 curated genes, was ranked by Pol II Ser5p occupancy at TSS in PTEN-WT HeLa cells and used in all the figure panels.

We re-analyzed PTEN ChIP-seq (SRR7902981, SRR7902982, SRR7902987) and Pol II Ser5p ChIP-seq (SRR7902985, SRR7902990) datasets from HeLa cells (GEO accession number GSE120478). We used our gene list (23,396 genes) curated from GRCh37/hg19 gene list (see Experimental Procedures for details) to generate heatmap and metagene plots. Our analysis shows PTEN occupancy at promoter proximal regions in thousands of genes as shown by metagene and heatmap plots (Figure 4A–4C). First, we ranked our gene list based on Pol II Ser5 promoter proximal signals (−50 to +200 bp from TSS) and used this sorted gene list to generate heatmaps. Interestingly, PTEN and Pol II Ser5p heatmaps showed a good overlap suggesting PTEN occupancy at promoter proximal regions correlates with Pol II Ser5p levels (Figure 4C, 4D). Surprisingly, Pol II Ser5p ChIP-seq shows increase in global Pol II Ser5p occupancy after PTEN loss in CRISPR-PTEN HeLa cells (Figure 4D–4E). Co-occupancy of PTEN with Pol II Ser5p and increases in Ser5 phosphorylation after PTEN loss corroborates our *in vitro* data that showed PTEN significantly dephosphorylates Pol II CTD at Ser5 position (Figure 3).

## DISCUSSION

PTEN overexpression and knock-down or knock-out resulting in thousands of differentially expressed genes have been known without much mechanism resolution. Until very recently, we and others have shown its role in maintaining Pol II pausing, a highly conserved phenomenon finetuning the transcriptional process [19], and PTEN occupancy at promoter proximal regions, at genome-wide scale, regulating gene expression [20]. Moving forward, our data presented here further corroborate these previous findings and provide novel insight of nuclear PTEN function. The association of PTEN with Pol II CTD phosphorylation status in cells (Figure 1) is an intriguing observation as RNA Pol II CTD is subject to extensive phosphorylation and dephosphorylation during the transcription cycle. Since specific post-translational modifications of Pol II CTD are linked to transcription initiation, elongation, and termination [17], the role of PTEN, perhaps as a phosphatase for Pol II CTD, has a wide impact on transcriptional regulation as well as co-transcriptional processes. Pol II CTD post-translational modifications play crucial roles in the regulation of Pol II elongation rate and alternative splicing [21–23]. Recently, Shen and co-workers demonstrated that PTEN interacts with the spliceosome and regulates alternative splicing [24]. Our data would support the possible role of PTEN in regulating co-transcriptional splicing due to its ability to modify Pol II CTD.

Biological General Repository for Interaction Datasets (BioGRID, https://thebiogrid.org) show that PTEN could have several hundred interacting partners, and its interaction with various nuclear factors has already been reported [6–8]. Our observation here that PTEN physically interacts with RNA Pol II, possibly as a dimeric form (Figure 2), lends both biological and clinical insight. Genetic and structure-based studies have provided evidence for PTEN dimerization, and the PTEN dimer is believed to be functionally active [21, 22]. Much of the pathophysiology of PTEN-related inherited and sporadic cancer and autism has been attributed to the canonical PTEN signaling pathway chief of which is the PI3K/AKT-mTOR pathway [23]. We add PTEN’s ability to dimerize and interact with, and dephosphorylate, RNA Pol II to the growing non-canonical roles. Given the ubiquitousness of PTEN and even more so, RNA Pol II, there will be far-reaching implications not only for overgrowth and malignancy, neurodevelopmental disorders such as autism, but also begin to explain human development as a whole and such disparate disorders as glucose metabolism, obesity and vascular remodeling [24–28].

TFIIH-associated kinase CDK7 phosphorylates Pol II Ser5 at the beginning of transcription process and the highest Ser5p peak is always observed at Pol II pause site (30–60 nt from TSS). As Pol II complex elongates after pause release, Pol II Ser5 phosphorylation is gradually removed. PTEN dephosphorylating Pol II CTD specifically at Ser5p (Figure 3) and the observed increase in genome-wide Pol II Ser5p occupancy (Figure 4D–4F) after PTEN loss by CRISPR provide further evidence of PTEN’s direct role in regulating global transcription. Pol II undergoes a rapid turnover at promoter proximal regions and less than 10% of Pol II complex reaches productive elongation [29]. It is unclear whether genome-wide PTEN occupancy at Pol II pause sites, which appear to decrease Pol II Ser5 phosphorylation level (Figure 4), contributes to premature termination of the Pol II complex, to productive elongation, or to both in a context-dependent manner. Egloff et al. reported that Pol II Ser7 phosphorylation is required for the recruitment of RPAP2 to snRNA genes that dephosphorylate Ser5p [30]. Remarkably, we observed that PTEN interacts with Pol II with the highest specificity for Ser7p (Figure 2A) and specifically dephosphorylates Pol II Ser5p (Figure 3) suggesting the requirement of Ser7p for PTEN to dephosphorylates Pol II Ser5p.

In conclusion, our data indicate that PTEN is a Pol II Ser5 phosphatase that directly binds with Pol II complex. Corroboratively, ChIP-seq analysis further provides evidence that PTEN dephosphorylates Pol II Ser5p at promoter proximal regions and has the ability to modulate global gene regulation. However, more work is needed to further build on this founding study specifically to understand how PTEN co-operates with other factors at promoter proximal regions to decide the fate of the Pol II complex, whether to terminate or to undergo productive elongation. The further understanding of PTEN-Pol II interactions will add a new dimension to understanding somatic and heritable PTEN-related carcinogenesis.

## MATERIALS AND METHODS

### Cell culture and reagents

BT-549 (HTB-122™) and MCF7 (HTB-22™) breast cancer cell lines were purchased from American Type Culture Collection (ATCC, Manassas, VA, USA) and molecularly authenticated by STRS analyses. Cells with early passage range 3–6 were used for the experiments. Both cell lines were cultured in DMEM/F-12 medium (Life Technologies, Grand Island, NY, USA) supplemented with 10% FBS (Thermo Scientific Gibco, Waltham, MA, USA) at 37° C with 5% CO_2_. To prepare PTEN expressing stable line, BT-549 (PTEN null) cells were transfected with pTet-Off (Clontech Takara, Mountain View, CA, USA) using Fugene^®^ 6 (Promega, Madison, WI, USA) according to the manufacturer’s protocols. Multiple stable clones were isolated using neomycin selection and tested with pTRE2Hyg-Luc according to manufacturer’s protocols for optimal expression and tetracycline regulation. A clone was transfected using Fugene^®^ 6 with a pTRE2Hyg (Clontech Takara) backbone containing 3X N-terminal FLAG-tagged PTEN or empty vector control. Both stable PTEN expressing and stable control clones were selected by hygromycin treatment. To prepare a PTEN knock-down stable line, MCF7 cells were transfected using Lipofectamine^®^ 2000 (Invitrogen, Carlsbad, CA, USA) with PTEN-targeting MISSION^®^ TRC shRNA TRCN0000002746 shRNA or the MISSION Non-targeting control SHC002 (both from Sigma-Aldrich, St. Louis, MO, USA). Stable clones were selected by puromycin treatment and clones with the best knockdown were used for the experiments. All the experiments were performed when cells were at 70–80% confluency.

### Expression and purification of PTEN protein

3xFLAG-tagged PTEN construct was designed with stable overexpression system generated as discussed above. BT-549 cells overexpressing 3x FLAG-tagged PTEN were grown in 5 × 15 cm dishes to ~90% confluency each, and cell pellets were collected. Protein lysate was prepared using the M-PER (Thermo Scientific) supplemented with protease inhibitor (Sigma-Aldrich). Cell lysates were incubated with anti-FLAG M2 agarose beads for 1 hour at 4° C followed by 5–6 times washing with pre-chilled TBS buffer containing 0.5M Tris-HCl (pH 7.5) and 1.5 M NaCl. PTEN protein was eluted by using elution buffer (50 mM Tris-HCl pH 7.5, 150 mA NaCl, 10 mM MgCl_2_, 0.1 mM DTT and 10% Glycerol) containing 3X FLAG peptide followed by clean-up of elution mix by using Pierce^TM^ centrifuge columns (Thermo Scientific Pierce).

### Immunoblotting and co-immunoprecipitation

Protein lysates were prepared using M-PER (Thermo Scientific) supplemented with protease and phosphatase inhibitors (Sigma-Aldrich) and quantified by using the BCA protein assay (Thermo Scientific). Lysates were separated by SDS-PAGE and transferred onto nitrocellulose membranes followed by probing with appropriate antibodies (see Supplementary Material for the list of antibodies). Blots were scanned digitally using the GE Amersham Imager 600 (GE Healthcare Life Science, Chicago, IL, USA). For immunoprecipitation, protein lysates were prepared using non-denaturing lysis buffer containing 20 mM Tris HCl pH 8, 137 mM NaCl, 1% NP-40, and 2 mM EDTA, supplemented with protease and phosphatase inhibitors (Sigma-Aldrich). Lysates were pre-cleared with Protein A/G Dynabeads™ (Thermo Scientific) followed by immunoprecipitation using appropriate antibodies. Immunoprecipitated proteins were immunoblotted and detected using the method described above.

### *In vitro* pull-down assay

200 ng of unphosphorylated or phosphorylated GST-CTD (prepared using CDK9/CyclinT1 as described above) were incubated with glutathione beads at 4° C for 3 hours followed by washing at room temperature to remove unincorporated GST-CTD. Beads were then incubated with purified PTEN at 4° C for 2 hours followed by stringent washings at room temperature. Beads were incubated at 100° C in the 2× Laemmli buffer for 5 minutes followed by immunoblotting.

### *In vitro* kinase and phosphatase assay

200 ng of GST-CTD (c-terminal domain of RNA Pol II) was incubated with CDK9/CyclinT1 (Abcam, Cambridge, MA, USA) for 2 hours at 30° C in kinase buffer containing 40 mM HEPES, pH 7.5, 10 mM MgCl2, 5 mM dithiothreitol, and 500 μM ATP (Invitrogen). Reaction was stopped by removing unincorporated ATP through a MicroSpin Column (GE Healthcare) according to the manufacturer’s protocol. PTEN phosphatase activity was tested by incubating phosphorylated GST-CTD with PTEN and Ssu72 (a positive control) in phosphatase buffer containing 50mM Tris-HCl, pH 6.5, 10 mM MgCl2, 20 mM KCl, and 5 mM DTT. After 2 hours of incubation at 30° C, reactions were stopped by adding 2× Laemmli buffer and incubated at 100° C for 5 minutes before immunoblotting.

### ChIP-seq data analysis

ChIP-seq raw data files were downloaded from Gene Expression Omnibus (GEO, accession number GSE120439) for PTEN ChIP-seq (SRR7902981, SRR7902982, SRR7902987) and Pol II Ser5p ChIP-seq (SRR7902985, SRR7902990). Reads were aligned against hg19 genome by bowtie2 using sensitive mode parameter (-D 15 -R 2 -N 0 -L 20 -i S,1,0.75). SAM files generated from Bowtie2 were converted into BAM files. BAM files were normalized by depth after removal of PCR duplicates and blacklisted regions. Heatmaps and metagene plots were generated in ngs.plot.r program using our curated gene list. GRCh37/hg19 gene list was curated after removing non-canonical, mitochondrial, genes shorter than 2 kb and overlapping genes on the same strand, which resulted in 21,396 genes. Pol II Ser5p ChIP-seq signals at promoter proximal regions (−50 bp to +200 bp form TSS) were counted by using bedcov function of SAMtools. The resultant gene list was sorted based on Pol II Ser5p promoter proximal signals and used for generating heatmaps.

## Abbreviations

CTD: C-terminal domain
ChIP-seq: Chromatin immunoprecipitation – sequencing
GEO: Gene expression omnibus
GRCh37: Genome reference consortium human build 37
shRNA: Short hairpin RNA
TSS: Transcription start site
TES: Transcription end site
